# Predictors for false-negative QuantiFERON-TB Gold assay results in patients with extrapulmonary tuberculosis

**DOI:** 10.1186/s12879-018-3344-x

**Published:** 2018-09-10

**Authors:** Youn Jeong Kim, Ji Young Kang, Sang Il Kim, Mee Soo Chang, Yang Ree Kim, Yeon Joon Park

**Affiliations:** 1 Division of Infectious disease, Department of Internal medicine, College of Medicine, The Catholic University of Korea, Seoul, Korea; 2 Division of Pulmonology, Department of Internal Medicine, College of Medicine, The Catholic University of Korea, Seoul, Korea; 3 Department of Pathology, Seoul National University Boramae Hospital, Seoul National University College of Medicine, Seoul, Korea; 4 Department of Laboratory medicine, College of Medicine, The Catholic University of Korea, Seoul, Korea

**Keywords:** IFN-gamma release assay, Extrapulmonary tuberculosis, False negative

## Abstract

**Backgrounds:**

Extrapulmonary tuberculosis (EPTB) is a heterogeneous disease, and diagnosis is sometimes difficult. We investigated the diagnostic performance of the QuantiFERON-TB Gold assay (QFT-GIT) according to sites of EPTB and predictors for false-negative QFT-GIT results.

**Methods:**

A total of 2176 patients were registered with active TB from January 2012 to December 2016 in Seoul St. Mary’s Hospital, a 1200-bed tertiary teaching hospital in Seoul, Korea. We retrospectively reviewed the medical records of 163 EPTB patients who underwent QFT-GIT.

**Results:**

False negative QFT-GIT results were found in 28.8% (95% CI 0.22–0.36) of patients with EPTB. In the proven TB group, negative QFT-GIT results were found in 28.6% (95% CI 0.04–0.71) of pleural, 8.3% 0.002–0.38of lymph node, 8.3% (95% CI 0.002–0.38) of skeletal and 5.8% (95% CI 0.001–0.28) of gastrointestinal TB cases. Among probable TB cases, QFT-GIT negative results were identified in 46.2% (95% CI 0.19–0.75) of skeletal, 33.3% (95% CI 10–0.65) of pericardial, 30.8% (95% CI 0.09–0.61) of pleural and 17.2% (95% CI 0.10–0.56) of gastrointestinal TB cases. In the possible TB cases, central nervous system TB (*n* = 21) was most frequent, and 66.7% (95% CI 0.43–0.85) of those showed QFT-GIT negative results. By multivariate analysis, possible TB was independently associated with false-negative QFT-GIT results (OR 4.92, 95% CI 1.51–16.06, *p* = 0.008).

**Conclusions:**

Prudent interpretation of QFT-GIT results might be needed according to anatomic site of involvement and diagnostic criteria in patients with high suspicion of EPTB.

## Background

Despite diagnostic and therapeutic advances, tuberculosis (TB) remains an important cause of morbidity and mortality. Early diagnosis and prompt treatment are crucial to reduce mortality and spread of TB. Although the incidence of TB in Korea has been decreasing over time, the incidence in 2016 was still estimated at 77 per 100,000 population [1]. In Korea, 6196 cases of extrapulmonary TB (EPTB) were reported among 30,892 new cases of TB at the end of 2016 [2]. EPTB is a heterogeneous disease involving various organs such as lymph nodes, bone, central nervous system (CNS), gastrointestinal system and genitourinary system, and the clinical manifestations of EPTB are varied. EPTB can be diagnosed by histopathologic features or clinical findings as well as microbiologic test. Diagnosis of EPTB is sometimes difficult because physicians do not always suspect the disease, have difficulty obtaining specimens, or do not perform appropriate diagnostic methods such as smear, polymerase chain reaction (PCR) or culture. The interferon (IFN)-γ release assay (IGRA) detects IFN-γ response to TB-specific antigens, early secretory antigen target-6 and culture filtrate protein-10, and has been used to support the diagnosis of latent or active TB [3, 4]. However, there is some controversy around the usefulness of IGRA for diagnosis of active TB, and it has not been clarified whether IGRA is useful for the diagnosis of EPTB [4]. Identification of factors associated with negative IGRA results in patients suspicious for EPTB may be helpful to optimize use of the assay in clinical practice. In this study, we investigated the diagnostic performance of QuantiFERON-TB Gold assay (QFT-GIT) according to categories and anatomic sites of EPTB, and identified predictors for false-negative assay results.

## Methods

### Study setting and population

A total of 2176 patients were registered with active TB from January 2012 to December 2016 in Seoul St. Mary’s Hospital, a 1200-bed tertiary teaching hospital in Seoul, Korea. We excluded patients aged < 18 years and those with pulmonary TB.

### Diagnostic definition

All patients were diagnosed based on clinical, histopathological, radiological and microbiological data. The clinical categories of patients with EPTB have been described previously [5–8]. Proven TB was defined as positive culture or polymerase chain reaction (PCR) results. Probable TB was defined if patients responded successfully to anti-TB medication and met one of following criteria: (1) histological findings in biopsy such as caseous granuloma; (2) body fluid such as cerebrospinal, pericardial, peritoneal or pleural fluid containing lymphodominant exudate with increased adenosine deaminase concentration; (3) unexplained urinary symptoms or persistent pyuria and radiological findings consistent with genitourinary TB; or (4) undocumented bacteria by tissue culture, and radiological findings with TB spondylitis or arthritis. Possible TB was defined if patients did not fulfill the above criteria but responded after completing anti-TB treatment.

### Interferon-gamma release assay (IGRA)

IGRA was performed using QuantiFERON-TB Gold in-tube in the department of laboratory medicine at Seoul St. Mary’s hospital according to manufacturer’s instructions (Cellestis, Carnegie, Victoria, Australia). Briefly, the QFT-GIT result was defined as positive if the IFN-γ level of Nil was < 8.0 IU/mL and that of TB antigen minus Nil was > 0.35 IU/mL and 25% of Nil value. Negative results was defined if the IFN-γ level of Nil was < 8.0 IU/mL, that of Mitogen minus Nil was > 0.5 IU/mL, and that of TB antigen minus Nil was < 0.35 IU/mL or 25% of Nil value. The results were reported as indeterminate if the IFN-γ level of Nil was < 8.0 IU/mL, that of TB antigen minus Nil was< 0.35 IU/mL or > 0.35 IU/mL and < 25% of Nil value, and Mitogen minus Nil was < 0.5 IU/mL (positive control failure) or if the IFN-γ level of Nil was > 8.0 IU/mL (negative control failure).

### Statistical analysis

The Student *t* test or the Mann–Whitney *U* test was used for analysis of continuous variables, and the χ^2^ test or Fisher’s exact test was used for categorical variables. Multivariate analysis using multiple logistic regression was performed for statistically significant predictors in the univariate analysis to determine the risk factors associated with false-negative QFT-GIT results. Statistical analysis was performed using SPSS 13.0 (SPSS Inc., Chicago, IL, USA), and *p* < 0.05 was considered statistically significant.

### Ethical approval

The study was approved by the Institutional Review Board of Seoul St. Mary’s Hospital(KC18RESI0142).

## Results

### Demographic characteristics

**Fig. 1:**
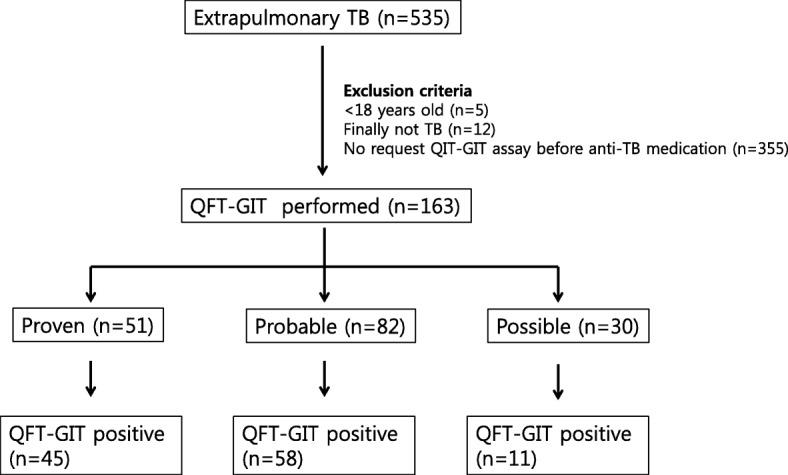
Study population. TB: tuberculosis; QFT-GIT: QuantiFERON-TB Gold assay

There were 535 patients with EPTB, and we excluded the following: five aged < 18 years, 12 who finally did not have TB, and 355 who did not undergo QFT-GIT before anti-TB medication. Finally, we included 163 patients with EPTB who were examined by QFT-GIT (Fig. 1). Fifty-one patients (31.2%) were classified as proven TB, 82 (50.3%) as probable TB, and 30 (18.4%) as possible TB. Gastrointestinal tract (*n* = 48) and skeletal system (*n* = 27) were the most common sites of EPTB presentation. CNS involvement was the third most common site of presentation (*n* = 26), followed by TB lymphadenopathy (*n* = 24), pleurisy without lung parenchymal involvement (*n* = 24), pericardium (*n* = 13), and others (*n* = 7). Disseminated TB was found in 5 patients. Median age was 51 years old, and 33.7% of patients were above 65 year old.

### Comparisons of patient characteristics according to QFT-GIT results

**Fig. 2:**
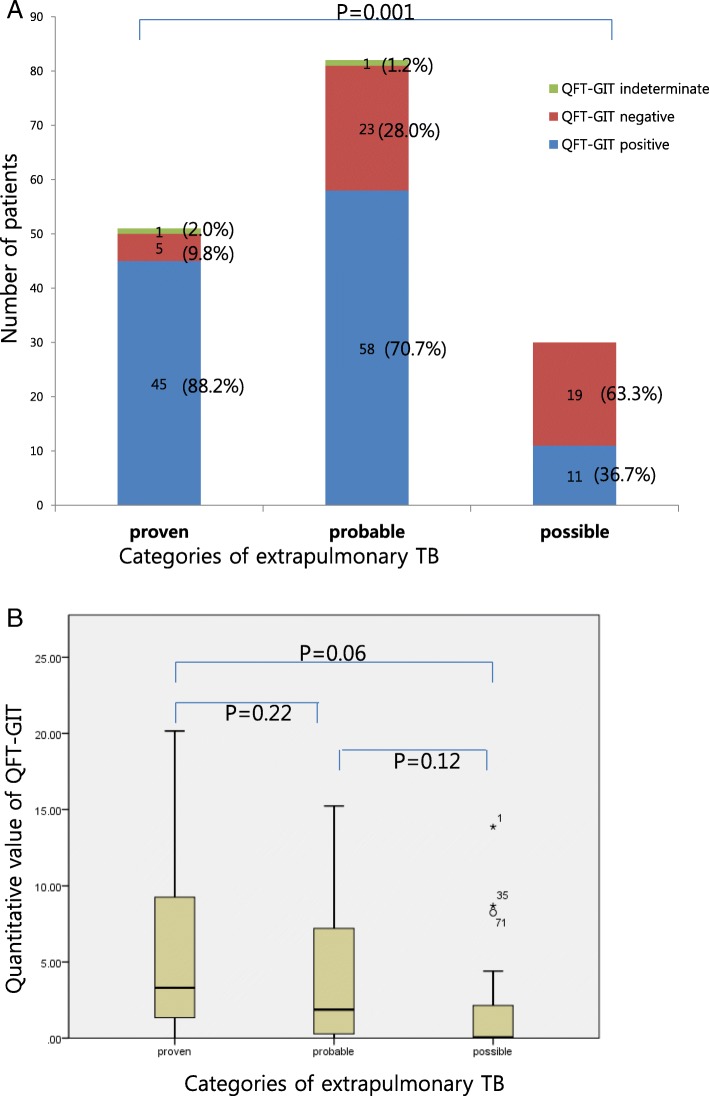
QFT-GIT results according to categories of extrapulmonary tuberculosis. **a** Patient proportion of QFT-GIT results according to categories of extrapulmonary tuberculosis. **b** Quantative value of QFT-GIT results according to categories of extrapulmonary tuberculosis

One hundred and sixty-three patients were examined by QFT-GIT assay, and 114 (69.9%) had a positive result and 2 (1.2%) an indeterminate result. Table 1 shows the comparison of demographic and laboratory characteristics according to QFT-GIT results in patients with EPTB. The median age was older in EPTB patients with positive QFT-GIT results than in those with negative results (median 55 [IQR 27.2, range 18–95] vs. 51[34, 22–83] years old, *p* = 0.09). Patients who had an old fibrotic scar on chest X-ray showed higher positive QFT-GIT results (15.8% [18/114] vs. 2.1% [1/47], *p* = 0.015). Laboratory findings such as white blood cell (WBC) count, total lymphocyte count, hemoglobin, erythrocyte sedimentation rate (ESR), C-reactive protein (CRP) and albumin did not differ according to QFT-GIT results. All patients with disseminated TB showed positive QFT-GIT results. The percentage of positive results was highest in the proven TB group (45/51, 88.2%), followed by 70.7% (58/82) in the probable TB group, and 36.6% (11/30) in the possible TB group (*p* = 0.001) (Fig. 2a). The median quantitative value of QFT-GIT was significantly higher in the proven TB group than in the possible TB group (3.3 vs. 0.05 IU/ml) (Fig. 2b). In the 51 proven cases, 5 cases (9.8%) showed negative QFT-GIT. The median quantitative value of QFT-GIT was significantly higher in the proven TB group than in the possible TB group (3.3 vs. 0.05 IU/ml) (Fig. 2b).

**Table 1:** Comparisons of patient characteristics and laboratory findings according to QFT-GIT results

	QFT-GIT (−), *n* = 47, n(%)	QFT-GIT (+), *n* = 114, n(%)	Indeterminate, *n* = 2, *n*(%)	*p* value^*^
Age, median, years (IQR, range)	51 (34, 22–83)	55 (27.2, 18–95)	66.5	0.09
Sex, male	25 (53.2%)	60 (52.6%)	0 (0%)	0.95
BMI, median, kg/m^2^ (IQR, range)	22.4 (4, 16.7–26.2)	22.1 (4.4, 19.2–25.7)	21.3	0.74
Underlying disease				
Diabetes mellitus	3 (6.4%)	13 (11.4%)	1 (50.0%)	0.33
Hypertension	13 (27.7%)	28 (25.6%)	1 (50.0%)	0.68
Malignancy	10 (21.3%)	22 (19.3%)	1 (50.0%)	0.19
Solid cancer	3	14	1	
Hematologic	7	8	0	
malignancy				
Chronic kidney disease	5 (10.6%)	7 (6.1%)	1 (50.0%)	0.32
Autoimmune disease	3 (6.4%)	2 (1.8%)	1 (50.0%)	0.12
Transplant	3 (6.4%)	6 (5.3%)	0 (0%)	0.78
HIV infection	0 (0%)	1 (0.9%)	0 (0%)	0.52
Prior tuberculosis history	2 (4.3%)	16 (14.0%)	0 (0%)	0.07
Old fibrotic scar on chest X-ray	1 (2.1%)	18 (15.8%)	0 (0%)	0.015
Disseminated disease	0 (0%)	5 (4.4%)	0 (0%)	0.33
White blood cells, × 10^9^/L, median (IQR, range)	66.6 (58.6,17.1–173)	58.2 (48.0–79.6,10.1–206.7)	67.5	0.39
Total lymphocyte, × 10^9^/L, median(IQR, range)	13.8 (13.2,2.6–49.2)	13.2 (10.2, 2.3–45.8)	7.5	0.62
Hemoglobin,g/dl, median (IQR, range)	11.75 (2.13, 7.4–28.20)	12.1 (3.2, 7.2–17.10)	8.85	0.36
ESR,mm/h, median (IQR, range)	46.0 (60.5, 2–120)	44.0 (54.4,3–120)	59.5	0.19
C-reactive protein, mg/dl, median (IQR, range)	1.87 (5.43,0.04–29.50)	2.03 (7.8,0.03–30.8)	1.11	0.79
Albumin, g/dl median(IQR, range)	3.5 (1.0,2.3–4.4)	3.5 (1.2,2.0–4.8)	2.9	0.52

^*^Intermediate value was excluded

*ESR* Erythrocyte sedimentation rate, *BMI* body mass index

### QFT-GIT results according to EPTB sites

**Fig. 3:**
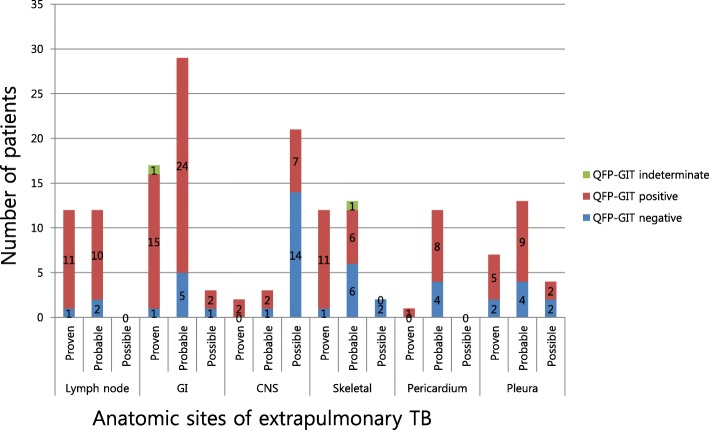
Patient proportion of QFT-GIT results according to categories of extrapulmonary tuberculosis in anatomic site. *GI* gastrointestinal tract. *CNS* central nervous system

Figure 3 shows the QFT-GIT results according to sites and categories of EPTB. In the proven TB group, negative QFT-GIT results were found in 28.6% (2/7) of pleura, 8.3% (1/12) of lymph node, 8.3% of skeletal and 5.8% (1/17) of gastrointestinal TB cases. In the probable TB group, negative QFT-GIT results were found in 46.2% (6/13) of skeletal, 33.3% (4/12) of pericardial, 30.8% (4/13) of pleural and 17.2% (5/17) of gastrointestinal TB cases. In the possible TB group, CNS TB (*n* = 21) was most frequent, and 14 of those (66.7%) had negative QFT-GIT results. Indeterminate QFT-GIT results were seen in 1 case of proven gastrointestinal TB and 1 case of probable skeletal TB. Patients with TB lymphadenopathy (18.4% [21/114] vs. 6.4% [3/47], *p* = 0.05) and gastrointestinal TB (36.0% [41/114] vs. 14.9% [7/47], *p* = 0.0008) had a higher percentage of positive QFT-GIT results. However, patients with CNS TB had a significantly lower percentage of positive QFT-GIT results (9.6% [11/114] vs. 31.9% [15/47], *p* = 0.0001).

### Risk factors associated with false-negative results of QFT-GIT

By univariate analysis, the predictors associated with false-negative QFT-GIT results were gastrointestinal TB (odds ratio [OR] 0.31, 95% confidence interval [CI] 0.12–0.75, *p* = 0.01), CNS TB (OR 4.38, 95% CI 1.83–10.51, *p* = 0.001), possible TB (OR 6.35, 95% CI 2.71–14.89, *p* = 0.0001), and old fibrotic scar on chest X-ray (OR 0.12, 95% CI 0.01–0.89, *p* = 0.03) (Table 2). By multivariate analysis, possible TB was independently associated with false-negative QFT-GIT results (OR 4.92, 95% CI 1.51–16.06, *p* = 0.008) (Table 2).

**Table 2:** Predictors associated with false-negative QFT-GIT results

	Univariate analysis*			Multivariate analysis*		
	OR	95% CI	*p*-value	OR	95% CI	*p*-value
Age > 65 years	0.50	0.23–1.09	0.08			
Prior tuberculosis history	0.27	0.06–1.23	0.09			
Peripheral lymphadenopathy	0.30	0.08–1.10	0.06			
Gastrointestinal TB	0.31	0.12–0.75	0.01	0.50	0.19–1.31	0.16
CNS TB	4.38	1.83–10.51	0.001	1.16	0.32–4.19	0.82
Possible TB	6.35	2.71–14.89	0.0001	4.92	1.51–16.06	0.008
Old fibrotic scar on chest X-ray	0.12	0.01–0.89	0.03	0.12	0.01–0.09	0.05

^*^Intermediate value was excluded in univariate and multivariate analysis

*CNS* central nervous system, *TB* tuberculosis

## Discussion

In Korea, the proportion of EPTB was 20.1% (*n* = 6196) of all cases of reported TB (*n* = 30,892) in 2016, which has slightly increased compared with 2009 (19.3%, 6923/35824) [2]. One study in the United States also showed that the proportion of EPTB increased from 1993 to 2006, and 18.7% were EPTB [9]. IGRA is a new immunologic diagnostic tool for active or latent TB based on T-cell immune response to TB antigen, which is not associated with non-tuberculous mycobacteria or BCG-vaccination [10, 11]. QFT-GIT assay uses an enzyme-linked immunosorbent assay to measure antigen-specific production of IFN-γ by circulating T cells in whole blood. The other test, the T-SPOT.TB (Oxford Immunotec, Oxford, United Kingdom), measures IFN- γ -spot-forming cells [11]. A meta-analysis showed that the sensitivity of QuantiFERON TB Gold and T-SPOT.TB for diagnosis of TB was 0.842 (95% CI 0.811–0.870) and 0.840 (95% CI 0.814–0.864)), respectively; specificity was 0.745 (95% CI 0.715–0.775) and 0.658 (95% CI 0.621–0.693), respectively; positive likelihood ratio was 3.652 (95% CI 2.180–6.117) and 2.196 (95% CI 1.727–2.794), respectively;and negative likelihood ratio was 0.212 (95% CI 0.109–0.414) and 0.246 (95% CI 0.161–0.377) respectively [12]. The role of IGRAs for diagnosis of active TB remains unclear. However, in real practice, IGRA may have an adjunctive role to diagnose TB or rule out active TB, especially if microbiological or pathological clues for TB cannot be found in patients with high clinical suspicion of TB. TB can be diagnosed by clinical features, radiology, pathology and microbiology. However, diagnosis of EPTB is more elusive than that of pulmonary TB because EPTB is not always suspected, and there is difficulty obtaining pathological evidence, and failure to perform appropriate diagnostic tests. Our study of EPTB cases showed that 28.8% of all cases and 9.8% of proven TB cases had negative QFT-GIT results. Among the proven and probable TB cases, there was a higher percentage of negative QFT-GIT result for pleural and skeletal TB. Our findings suggest that physicians should not rule out the possibility of TB even though suspicious skeletal or pleural TB cases show negative QFT-GIT results.

Previous studies have shown that the quantitative response of IGRA is higher in active TB than in latent TB, and generally falls during anti-TB medication [13, 14]. It is not clear whether a high response of IGRA is associated with disease burden and is useful for diagnosis of active TB [15]. In our study, quantitative values for QFT-GIT were higher in proven than in possible cases of TB, although the difference was not significant. All cases of disseminated TB had positive QFT-GIT results. This supports the diagnostic utility of quantitative values of IFN-γ for patients with severe TB infection, however, further, large studies are needed to confirm this.

Patients with CNS involvement showed a higher percentage of negative QFT-GIT assay results than those with TB involving other sites. In cases of TB meningitis, sensitivity of conventional diagnostic tests such as acid-fast bacilli stain are reported to be as low as 30%, and PCR-based assays have the disadvantage of low sensitivity [16, 17]. Mycobacterial culture of CSF provides definitive diagnosis for patients with TB meningitis, however, culture requires long incubation of up to 8 weeks. Because of high mortality and neurologic sequelae, especially in highly TB endemic areas, clinically suspicious TB meningitis should be treated promptly before a definitive diagnosis is obtained [18]. In our practice, possible cases accounted for 80.7% of CNS TB patients, and this may have affected our finding that possible TB was independently associated with false-negative QFT-GIT assay results. Some studies have shown that sensitivity of IGRA is affected by HIV status, diabetes mellitus, neutropenia and immunosuppression [15, 19–22]. Our present study also showed that patients with positive QFT-GIT assay were older than those with negative results, although this difference was not significant. In Korea, which is an intermediate TB endemic area, older people have more chance of being exposed to TB, and the IGRA positive rate increases with age [23, 24]. Mori et al. reported that the higher QFT-GIT positivity rates were found in elderly people, and specific IFN- γ response may wane considerably with time after infection [25]. Our study included many elderly people of 33.7% of above 65 years old, and this age distribution may underestimate the false negative QFT-GIT results. In contrast to previous studies, we did not identify underlying disease or neutropenia as a risk factor for false-negative IGRA results.

Our study had some limitations. First, the number of patients according to EPTB organs or categories was not even because this was a single center retrospective study. Nevertheless, our study included relatively a large number of EPTB cases and analyzed performance of QFT-GIT according to EPTB site or diagnostic category. Second, some may argue probable EPTB cases, however our study can reflect real practice in intermediate TB endemic area.

## Conclusions

In conclusion, false negative QFT-GIT results were found in 28.8% of patients with EPTB, and the anatomic site of TB may affect the positive rate of QFT-GIT results. The possibility of TB should be considered especially in patients with clinical suspicion of pleural or bone TB although QFT-GIT results are negative. Prudent interpretation of QFT-GIT might be needed according to site of involvement and diagnostic criteria in patients with high suspicion of EPTB.

## Abbreviations

CNS: Central nervous system
EPTB: Extrapulmonary tuberculosis
IGRA: Interferon-gamma release assay
GI: Gastrointestinal
PCR: Polymerase chain reaction
QFT-GIT: QuantiFERON-TB Gold assay
TB: Tuberculosis
